# The complete plastome of the South African species, *Amaryllis belladonna* L. (Amaryllidaceae)

**DOI:** 10.1080/23802359.2021.1997121

**Published:** 2021-11-12

**Authors:** Zoë Dennehy, Jordan Bilsborrow, Alastair Culham, John David, Kálmán Könyves

**Affiliations:** a School of Biological Sciences, University of Reading, Reading, United Kingdom; b Royal Horticultural Society, RHS Garden Wisley, Wisley, United Kingdom

**Keywords:** *Amaryllis*, Amaryllidoideae, Cape Province, geophyte, ornamental plant

## Abstract

The complete plastome sequence of *Amaryllis belladonna* L. is assembled and annotated. This is the type species of *Amaryllis* and therefore the type of the family Amaryllidaceae and as such, is important to document the phylogenetic position of the family. The plastome sequence has a length of 158,145 bp, with the large single copy (LSC) regions comprising 85,963 bp, the small single copy (SSC) 18634 bp and two identical inverted repeats (IR) regions each of 26,774 bp. Phylogenetic analysis fully resolved *Amaryllis* in a clade with *Crinum* L. in the Amaryllidoideae, as expected, with the Allioideae as a sister group. *Agapanthus* (Agapanthoideae) is a sister to the other two subfamilies in the Amaryllidaceae. The phylogenetic tree produced corresponds to previous topologies based on plastome molecular markers including *matK*, *ndhF* and *rbcL*. This is the first paper reporting the whole plastome comparison of the type genera of all three subfamilies in the Amaryllidaceae.

The asparagalean family Amaryllidaceae J. St.-Hil. consists of three subfamilies: Agapanthoideae Endl. (one genus with nine species), Allioideae Herb. (13 genera with 795 species) and Amaryllidoideae (75 genera with ∼900 species; Meerow et al. 2020). The family has a cosmopolitan distribution, with centers of diversity for the Amaryllidoideae in the Mediterranean Basin, South Africa, and South America (Rønsted et al. 2012). The relationship between the subfamilies: (Agapanthoideae, Allioideae, Amaryllidoideae) is well-established based on plastid DNA data (Fay et al. 2000; Pires et al. 2006; Seberg et al. 2012; Givnish et al. 2018), however, the relationship between genera within the Amaryllidoideae remains elusive (Meerow et al. 2003; Meerow et al. 2006; Garcia et al. 2014). *Amaryllis belladonna* is the type species of the genus *Amaryllis* L. and consequently the type of the family Amaryllidaceae. *Amaryllis* comprises two species, both native to the Cape Province in South Africa. *Amaryllis belladonna*, also known as the ‘belladonna lily’, is a widely planted ornamental of economic importance (Tallini et al. 2017) and may have antiplasmodial uses (Cho et al. 2018). *Amaryllis paradisicola* Snijman is a rare species from Richtersveld National Park (Snijman and Williamson 1998) comprising, possibly, around 2000 individuals (Snijman et al. 2016). Here we report the complete plastome sequence of *A. belladonna* to contribute to the bioinformatic and systematic knowledge of the Amaryllidaceae.

Scape tissue was collected in silica gel from *A. belladonna* blooming at the Royal Horticultural Society’s Garden, Wisley, UK (51.312695° N, 0.476724° W). A herbarium voucher was deposited at WSY (WSY0150055; contact: Yvette Harvey, YvetteHarvey@rhs.org.uk). Total genomic DNA was extracted using the Qiagen DNeasy Plant Mini Kit (QIAGEN, Manchester, UK). Library development and Illumina HiSeq 150 bp PE sequencing were completed by Novogene Company Limited (Cambridge, UK).

Fast-Plast v1.2.8 (McKain and Wilson 2017) and NovoPlasty v3.7.0 (Dierckxsens et al. 2017) were used to assemble the plastome. The Bowtie reference index was built using Asparagales plastomes included in Fast-Plast. The complete plastome of *Narcissus poeticus* L. (MH706763; Könyves et al. 2018) was used as the starting seed for the NovoPlasty assembly. The memory was limited to 6 Gb. The complete plastome was annotated against the published *Narcissus poeticus* L. plastome (MH706763; Könyves et al. 2018) using Geneious Prime (v2020.2.5; https://www.geneious.com/). The *A. belladonna* plastome sequence was aligned to 20 published Asparagales plastome sequences including *Hyacinthoides non-scripta* (L.) Chouard ex Rothm. (MN824434, Asparagaceae; Garnett et al. 2020) and *Xanthorrhoea preissii* Endl. (KX822774, Asphodelaceae) as outgroups using the default settings with MAFFT v7.450 (Katoh et al. 2002; Katoh and Standley 2013) in Geneious Prime. Poorly aligned regions of the total alignment were excluded using trimAI v1.2 with default settings and converted to a FASTA file using readAI v1.2 (Capella-Gutiérrez et al. 2009). A maximum likelihood estimation was performed with RAxML v8.2.11 (Stamatakis 2014) in Geneious Prime using the model GTR + G + I and 1000 bootstrap replicates.

The plastome sequence of *A. belladonna* (MZ433380) is 158,145 bp in length, comprising the LSC (85,963 bp), SSC (18,634 bp) and two inverted repeat regions each 26,774 bp. The plastome contains 86 protein-coding, 38 tRNA and eight rRNA genes. Of these eight protein-coding genes, eight tRNA genes and four rRNA genes are duplicated in the inverted repeats. There were high levels of gene synteny between *A. belladonna* and other published Amaryllidoideae plastomes (Könyves et al. 2018; Li et al. 2018; Zhang et al. 2020). The phylogenetic tree produced corresponds to previous topologies based on plastome molecular markers including *matK* (Ito et al. 1999; Rønsted et al. 2012; Chen et al. 2013), *ndhF* and *rbcL* (Chen et al. 2013). Comparison within Amaryllidoideae samples confirms that this subfamily has a lower substitution rate than Allioideae.

The position of *A. belladonna* in the phylogenetic tree is congruent with previous papers (Ito et al. 1999; Rønsted et al. 2012; Chen et al. 2013) and the substitution rate modeled on the tree is similar to that for other Amaryllidoideae, and lower than that in Allioideae (Figure 1). As the type species of the family Amaryllidaceae and the subfamily Amaryllidoideae, this is a new and important reference in the plastome-based phylogeny of the group.

**Figure 1.**
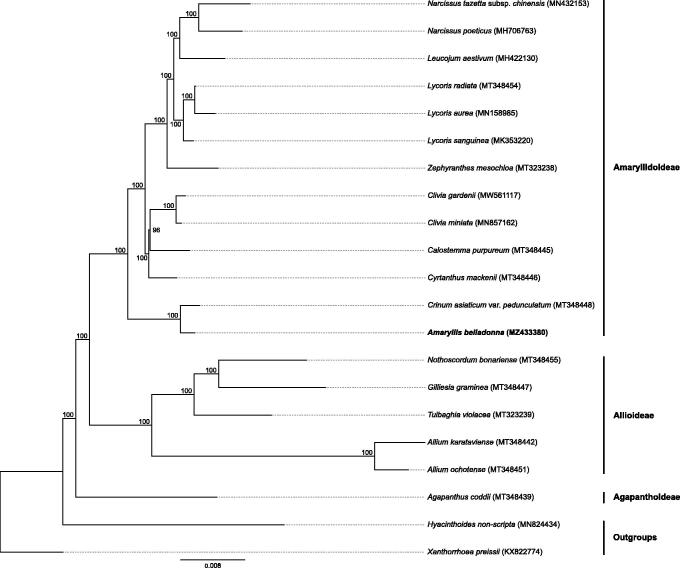
RaxML phylogenetic tree based on 21 asparagalean complete plastome sequences. Bootstrap support values are shown at each branch and GenBank accession numbers are given in brackets, subfamilies of the samples are shown on the right. Text in bold shows the plastome assembled in this study.

## Data Availability

The annotated plastome sequence is available on GenBank on NCBI (https://www.ncbi.nlm.nih.gov) under accession number MZ433380. Raw sequence data were deposited in SRA (BioProject: PRJNA743246); SRA: SRR15031247; BioSample: SAMN20014353).

## References

[CIT0001] Capella-Gutiérrez S, Silla-Martínez JM, Gabaldón T. 2009. trimAL: a tool for automated alignment trimming in large-scale phylogenetic analyses. Bioinformatics. 25(15):1972–1973. 19505945 10.1093/bioinformatics/btp348 PMC2712344

[CIT0002] Chen S, Kim D-K, Chase MW, Kim J-H. 2013. Networks in a large-scale phylogenetic analysis: reconstructing evolutionary history of Asparagales (Lilianae) based on four plastid genes. PLoS One. 8(3):e59472. 23544071 10.1371/journal.pone.0059472 PMC3605904

[CIT0003] Cho N, Du Y, Valenciano AL, Fernández-Murga ML, Goetz M, Clement J, Cassera MB, Kingston DG. 2018. Antiplasmodial alkaloids from bulbs of *Amaryllis belladonna* Steud. Bioorg Med Chem Lett. 28(1):40–42. 29162457 10.1016/j.bmcl.2017.11.021 PMC5753767

[CIT0004] Dierckxsens N, Mardulyn P, Smits G. 2017. NOVOPlasty: de novo assembly of organelle genomes from whole genome data. Nucleic Acids Res. 45(4):e18. 28204566 10.1093/nar/gkw955 PMC5389512

[CIT0005] Fay MF, Rudall PJ, Sullivan S, Stobart KL, De Bruijn AY, Reeves G, Qamaruz-Zaman F, Hong WP, Joseph J, Hahn WJ, et al. 2000. Phylogenetic studies of Asparagales based on four plastid regions. In: Wilson KL, Morrison DA, editors. Melbourne, Australia: Monocots: Systematics and Evolution, CSIRO; p. 360–371.

[CIT0006] Garcia N, Meerow AW, Soltis DE, Soltis PS. 2014. Testing deep reticulate evolution in amaryllidaceae tribe hippeastreae (Asparagales) with ITS and chloroplast sequence data. Syst Bot. 39(1):75–89.

[CIT0007] Garnett GJL, Könyves K, Bilsborrow J, David J, Culham A. 2020. The complete plastome of *Hyacinthoides non-scripta* (L.) Chouard ex Rothm. (Asparagaceae). Mitochondrial DNA B Resour. 5(1):1003–1004. 33366847 10.1080/23802359.2020.1720543 PMC7748465

[CIT0008] Givnish TJ, Zuluaga A, Spalink D, Gomez MS, Lam VKY, Saarela JM, Sass C, Iles WJD, de Sousa DJL, Leebens-Mack J, Pires JC, et al. 2018. Monocot plastid phylogenomics, timeline, net rates of species diversification, the power of multi-gene analyses, and a functional model for the origin of monocots. Am J Bot. 105(11):1888–1910. 30368769 10.1002/ajb2.1178

[CIT0009] Ito M, Kawamoto A, Kita Y, Yukawa T, Kurita S. 1999. Phylogenetic relationships of Amaryllidaceae based on *matK* sequence data. J Plant Res. 112(2):207–216.

[CIT0010] Katoh K, Misawa K, Kuma K, Miyata T. 2002. MAFFT: a novel method for rapid multiple sequence alignment based on fast Fourier transform. Nucleic Acids Res. 30(14):3059–3066. 12136088 10.1093/nar/gkf436 PMC135756

[CIT0011] Katoh K, Standley DM. 2013. MAFFT multiple sequence alignment software version 7: improvements in performance and usability. Mol Biol Evol. 30(4):772–780. 23329690 10.1093/molbev/mst010 PMC3603318

[CIT0012] Könyves K, Bilsborrow J, David J, Culham A. 2018. The complete chloroplast genome of *Narcissus poeticus* L. (Amaryllidaceae: Amaryllidoideae). Mitochondrial DNA Part B. 4(2):3364–3365. 10.1080/23802359.2018.1521311 PMC6390724 30854464

[CIT0013] Li M-DL, Wu X, Wu J-J, Zhou X, Wang R-H, Qi Z-C. 2018. Characterization of the complete chloroplast genome of summer snowflake (*Leucojum aestivum*, Amaryllidaceae). Mitochondrial DNA B Resour. 3(2):1069–1070. 33474418 10.1080/23802359.2018.1501309 PMC7799984

[CIT0014] McKain M, Wilson M. 2017. Fast-Plast: rapid de novo assembly and finishing for whole chloroplast genomes. https://github.com/mrmckain/Fast-Plast.

[CIT0015] Meerow AW, Francisco-Ortega J, Kuhn DN, Schnell RJ. 2006. Phylogenetic relationships and biogeography within the Eurasian clade of Amaryllidaceae based on plastid *ndhF* and nrDNA ITS sequences: lineage sorting in a reticulate area? 31(1):42–60.

[CIT0016] Meerow AW, Gardner EM, Nakamura K. 2020. Phylogenomics of the Andean tetraploid clade of the American Amaryllidaceae (subfamily Amaryllidoideae): unlocking a polyploid generic radiation abetted by continental geodynamics. Front Plant Sci. 11:582422. 33250911 10.3389/fpls.2020.582422 PMC7674842

[CIT0017] Meerow AW, Lehmiller DJ, Clayton JR. 2003. Phylogeny and biogeography of Crinum L. (Amaryllidaceae) inferred from nuclear and limited plastid non-coding DNA sequences. Bot J Linnean Soc. 141(3):349–363.

[CIT0018] Pires C, Maureira I, Givnish T, Systma K, Seberg O, Peterson G, Davis J, Stevenson D, Rudall P, Fay M, et al. 2006. Phylogeny, genome size, and chromosome evolution of asparagales. ALISO. 22(1):287–304.

[CIT0019] Rønsted N, Symonds MRE, Birkholm T, Christensen SB, Meerow AW, Molander M, Molgaard P, Petersen G, Rasmussen N, van Staden J, et al. 2012. Can phylogeny predict chemical diversity and potential medicinal activity of plants? A case study of Amaryllidaceae. BMC Evol Biol. 12:182. 22978363 10.1186/1471-2148-12-182 PMC3499480

[CIT0020] Seberg O, Petersen G, Davis JI, Pires JC, Stevenson DW, Chase MW, Fay MF, Devey DS, Jørgensen T, Sytsma KJ, et al. 2012. Phylogeny of the Asparagales based on three plastid and two mitochondrial genes. Am J Bot. 99(5):875–889. 22539521 10.3732/ajb.1100468

[CIT0021] Snijman DA, Van Wyk PCV, Raimondo D, von Staden L. 2016. *Amaryllis paradisicola.* The IUCN red list of threatened species 2016: e.T103633495A104109638; [accessed 2021 July 16]. 10.2305/IUCN.UK.2016-3.RLTS.T103633495A104109638.en

[CIT0022] Snijman DA, Williamson G. 1998. A new species of *Amaryllis* from the Richtersveld, South Africa. Bothalia. 28(2):192–196.

[CIT0023] Stamatakis A. 2014. RAxML version 8: a tool for phylogenetic analysis and post-analysis of large phylogenies. Bioinformatics. 30(9):1312–1313. 24451623 10.1093/bioinformatics/btu033 PMC3998144

[CIT0024] Tallini LR, de Andrade JP, Kaiser M, Viladomat F, Nair JJ, Zuanazzi JAS, Bastida J. 2017. Alkaloid constituents of the Amaryllidaceae plant *Amaryllis belladonna* L. Molecules. 22(9):1437. 10.3390/molecules22091437 PMC6151567 28858260

[CIT0025] Zhang F, Wang T, Shu X, Wang N, Zhuang W, Wang Z. 2020. Complete chloroplast genomes and comparative analyses of *L. chinensis*, *L. anhuiensis*, and *L. aurea* (Amaryllidaceae). IJMS. 21(16):5729. 10.3390/ijms21165729 PMC7461117 32785156

